# The effect of fertility-sparing surgery on sexuality and global quality of life in women with malignant ovarian germ cell and sex cord stromal tumors: an analysis of the CORSETT database of the AGO study group

**DOI:** 10.1007/s00404-021-06019-5

**Published:** 2021-07-21

**Authors:** Annette Hasenburg, Hellmut Plett, Bernhard Krämer, Elena Braicu, Bastian Czogalla, Michaela Bossart, Susanne Singer, Doris Mayr, Annette Staebler, Andreas du Bois, Stefan Kommoss, Theresa Link, Alexander Burges, Florian Heitz, Jacqueline Keul, Fabian Trillsch, Philipp Harter, Pauline Wimberger, Paul Buderath, Maximilian Klar

**Affiliations:** 1 Department of Gynecology and Obstetrics, University Medical Center Mainz, Mainz, Germany; 2 Department of Gynecology and Gynecologic Oncology, Ev. Kliniken Essen-Mitte, Essen, Germany; 3 Department for Women’s Health, Tuebingen University Hospital, Tuebingen, Germany; 4 Department of Gynecology, Campus Virchow Clinic, Charité Berlin, Berlin, Germany; 5 Department of Obstetrics and Gynecology, University Hospital, LMU Munich, Munich, Germany; 6 Department of Gynecology and Obstetrics, University Medical Center Freiburg, Freiburg, Germany; 7 Division of Epidemiology and Health Care Research, University Medical Center Mainz, Institute of Medical Biostatistics, Epidemiology and Informatics, Mainz, Germany; 8 Institute of Pathology, University Hospital, LMU Munich, Munich, Germany; 9 Division of Gynecologic Pathology, Institute of Pathology and Neuropathology, University of Tübingen, Tuebingen, Germany; 10 Department of Gynecology and Obstetrics, Medical Faculty, University Hospital Carl Gustav Carus, Technische Universität Dresden, Dresden, Germany; 11 Department of Gynecology and Obstetrics, University Hospital Essen, Essen, Germany; 12 University Cancer Centre Mainz, Mainz, Germany

**Keywords:** Ovarian germ cell tumors, Sex cord stromal tumors, Fertility-sparing surgery, Sexuality, Quality of life

## Abstract

**Purpose:**

Malignant ovarian germ cell (MOGCT) and sex cord stromal tumors (SCST) are ovarian neoplasms that affect disproportionally young women. Little is known about the impact of surgical and adjuvant management of these patient’s sexual life. This study investigated the effect of fertility-sparing surgery on sexual activity and global quality of life (gQoL) in women with MOGCT and SCST.

**Methods:**

CORSETT was an observational, multicenter, mixed retrospective/prospective cohort study of the AGO study group. Women of any age who had been diagnosed with MOGCTs and SCSTs between 2001 and 2011 were asked to complete the Sexual Activity Questionnaire (SAQ) and the EORTC QLQ-C30.

**Results:**

In total, 355 patients were included. Of these, 152 patients with confirmed histological diagnosis had completed the questionnaires. A total of 106 patients were diagnosed with SCST and 46 with MOGCT.

Totally, 83 women (55%) were sexually active. After fertility-sparing surgery, patients had a 2.6 fold higher probability for being sexually active than after non-fertility-conserving treatment (unadjusted odds ratio (OR) 2.6, *p* = 0.01). After adjustment for age, time since diagnosis, FIGO stage, histology and phase of disease, the OR dropped to 1.8 (*p* = 0.22).

Of the sexually active patients, 35 (42%) reported high levels of discomfort during intercourse; 38% after fertility-sparing; and 58% after non-fertility-sparing surgery (adjusted OR 2.8, *p* = 0.18).

Women with fertility-conserving treatment reported a significantly better global QoL (*F*_adj_ 2.1, 6.2 points difference, *p* = 0.03) but not more pleasure during intercourse than women without fertility-sparing surgery (*F*_adj_ 0.4, *p* = 0.52).

**Conclusion:**

Fertility preserving approaches should be offered to every patient, when oncologically acceptable.

## Introduction

### Purpose

Malignant ovarian germ cell (MOGCT) and sex cord stromal tumors (SCST) are rare ovarian neoplasms that, however, affect a disproportionally high number of young patients. In a global population-based study on cancer of the ovaries, non-epithelial malignant tumors comprised approximately 5–6% of all ovarian malignancies in Europe [1].

MOGCT represents 2–3% of ovarian malignancies [2]. They primarily arise in young women between 10 and 30 years of age and represent 70 percent of ovarian tumors in this age group [3]. With an incidence peak in childbearing age, the majority of women may wish to retain their reproductive potential.

The goal of surgery for MOGCT is complete tumor resection and adequate staging. The oncological safety of fertility-preserving surgery for women with early stage disease has been extensively evaluated and is currently the gold standard [2, 4, 5]. There is no evidence for the benefit of hysterectomy or lymphadenectomy [6]. According to the European Society of Medical Oncology (ESMO), a fertility-retaining approach is indicated for every woman with MOGCT wishing to retain her reproductive potential, even with advanced disease [7, 8]. In a retrospective study of a single center in Korea, pregnancy rate reached 75% and live birth rate 65% after fertility-sparing surgery [9].

A distinct characteristic of MOGCT is the high sensitivity to chemotherapy [5]. Although there is a lack of randomized controlled trials for patients with MOGCTs, chemotherapy is considered necessary for patients beyond stage IA. Therapy should be platinum based and include additionally etoposid [6]. As a third agent, bleomycin or ifosfamid may be added for a total therapeutic length of four cycles [6]. With this combination of fertility-sparing surgery and combination chemotherapy, 5 year overall survival was high with up to 97% [8, 9]. The 5 year disease free survival for all stages was reported with 86% [9].

SCSTs are neoplasm of the ovary that represent approximately 1.2 percent of all primary ovarian tumors and are mainly diagnosed in women with a median age around 50 years [8, 10]. Surgery is required for staging, and therapy should consist of a median lower laparotomy, inspection and palpation of the abdomen, peritoneal cytology, and tumor removal through salpingo-oophorectomy. Granulosa cell tumors, Sertoli–Leydig cell tumors (SLCTs) of G2/G3 grading and steroid cell tumors require surgical treatment analogous to ovarian cancer staging. In case of granulosa cell tumors, hysteroscopy and curettage are recommended because of an increased risk of endometrial cancer. The role of regional lymph node removal is unclear. For young patients, fertility-sparing surgery is an option. The benefit of adjuvant chemotherapy also remains uncertain; some patients with FIGO IC disease or with remaining tumor may benefit from platinum-containing protocols [6]. In a study evaluating incidence and survival for patients with non-epithelial ovarian cancer between 1978 and 2016 in Denmark, the 5 year relative survival of SCST was reported around 74% [8]. It was lowest in the early study period (51%) and increased during time up to 90%. This was explained by centralization of treatment and improvement in management of the disease. Besides calendar year of diagnosis, overall mortality was associated with age and stage [8].

Fertility-sparing surgery for cancer patients has two goals: the option to preserve childbearing and the option to preserve hormone production. Up to now there is very limited information on how sexuality and quality of life may be changed by the surgical approach (fertility sparing or non-fertility sparing) and the subsequent systemic therapy.

Sexual activity and functioning are important factors influencing quality of life. Immediately after diagnosis of cancer, most patients focus on anticancer treatment and its challenges and sexuality becomes less important [11]. In this scenario, the primary objective of treatment is to cure the disease or prolongation of life and maintenance of quality of life [12]. Since survival rates and life expectancy in patients with MOGCTs and SCTs are excellent, quality of life concerns and the underlying factors move into focus of survivorship. However, despite a high prevalence of sexual dysfunction in patients with gynecologic cancers, awareness of health-care providers to this need is limited [13].

After salpingo-oophorectomy, postmenopausal patients may suffer from loss of libido while premenopausal patients possibly suffer from premature onset of menopause. The stromal and the hilar interstitial cells in the ovaries are essential for the synthesis of androgens (testosterone and androstendione), which, among other factors, preserve a woman’s libido even after menopause [14, 15]. Serum testosterone levels do not vary after natural menopause. However, women 55 years or older who underwent bilateral salpingo-oophorectomy (BSO) have significantly lower testosterone levels than women of the same age group who did not receive BSO [16]. Therefore, non-fertility-sparing surgery can impact libido in all age groups.

Additionally, women undergoing non-fertility-sparing surgery may suffer from hot flashes, vaginal dryness, dyspareunia, decreased sexual desire, decreased ability to achieve orgasm, loss of sensation in the genital area, anxiety, depression changes in self- and body-image, and interpersonal relationship-changes with their partner [17].

In a cross-sectional study of 189 ovarian cancer survivors and 287 age-adjusted healthy controls, cancer patients were sexually less active (47 vs. 53%) [18]. Sexually active ovarian cancer patients reported lower levels of sexual pleasure (*p* < 0.001) and higher levels of discomfort (*p* < 0.001) than controls. Lack of interest and physical problems were significantly more common in sexually inactive cancer patients compared to controls [18].

In our study, we aimed to investigate the prevalence of fertility-sparing treatment and systemic therapy for patients with MOGCTs and SCTSs among German centers of the AGO (Arbeitsgemeinschaft Gynäkologische Onkologie) study group and the impact of the surgical technique—either fertility sparing or non-fertility sparing—on quality of life and sexuality.

## Methods

The Current Ovarian geRm cell and SEx cord stromal Tumour Treatment strategies (CORSETT) study was an observational, multicenter, mixed retrospective/prospective cohort study of the AGO study group. Women of any age who had been diagnosed with MOGCTs and SCSTs or dermoid cysts with immature/malignant somatic components between 2001 and 2011 were contacted by each center and consented to the study (ethical approval: 513/13 Freiburg).

They were asked to complete questionnaires to evaluate QoL (EORTC QLQ-C30) and sexuality (Sexual Activity Questionnaire (SAQ) Fallowfield). The SAQ was used to assess sexual functioning in terms of habit, pleasure and discomfort during sexual intercourse [19]. The questionnaire is divided into three sections: In section “Introduction”, patients are asked about their sexual relationship and whether they are sexually active or not. Those who are not sexually active, go on to complete section “Results” and omit section “Discussion”. In Sections “Results”, possible reasons for sexual inactivity are listed and patients can choose those that apply to them. Additional space for any personal reasons is provided. Section 3 consists of 10 questions assessing those aspects of sexual functioning that may be influenced by hormonal status like desire, frequency, satisfaction, dryness of the vagina and penetration pain [19]. For all the 3 subscales, habit (range 0–3), discomfort (range 1–6) and pleasure (range 0–18), higher values indicate more agreeable outcomes. Discomfort was evaluated as pain during sexual intercourse and dryness of the vagina [19]. From the EORTC QLQ-C30, we used its global quality of life scale.

Before conducting the statistical analysis, a detailed analysis plan was developed by a team of clinicians and quality of life experts. The analyses included a description of the sample characteristics and the proportion of sexually active women. Among those who were not active, the proportion of patient-reported reasons for non-activity was calculated. Among the sexually active, the distribution of pleasure and discomfort during sexual intercourse scores was investigated using histograms. As discomfort was not normally distributed, it was dichotomized based on its median (scores 0–4.9 = high discomfort, scores 5–6 = little discomfort). For the endpoints sexual activity and discomfort, the odds ratios (OR) and 95% confidence intervals (CI) comparing fertility-sparing with non-fertility-sparing surgery were calculated using multivariate logistic regression models. The effect of type of surgery on the endpoints pleasure and global QoL was investigated using multivariate analysis of variance.

In all models, we adjusted for age at the time of the survey, histology, time since diagnosis, FIGO stage and recurrence (yes vs. no).

## Results

### Sample description

Out of 355 MOGCT and SCSCT patients, 168 (47.3%) had completed the Sexual Activity Questionnaire (SAQ) and were thus included into the study; 65 of those who did not complete the SAQ were not actively declining but had not received the questionnaire; 106 participants were diagnosed with SCST, 46 with MOGCT and 16 with unknown histology (Table 2), resulting in 152 patients to be included in the analysis.

The participants who had completed the SAQ were younger (average age 50 vs 53 yrs.) and less likely had experienced disease recurrence than the 187 participants of the CORSETT database who had not completed the SAQ (*p* < 0.001) (Table 1).

**Table 1:** Comparison of patients who fulfilled the inclusion criteria and completed or not completed the SAQ

Basic characteristics	SAQ not completed		SAQ completed		*p* value
	*n* = 187		*n* = 168		
	*N*	%	*N*	%	
Age at interview					
Average	53		50		
Minimum–maximum	17–100		17–86		0.15
Histology					
SCST	153	82%	106	63%	< 0.001
MOGCT	31	17%	46	27%	
Unknown	3	2%	16	10%	
Surgical method					
Fertility sparing (laparoscopy or laparotomy)	88	47%	87	52%	0.04
Non-fertility sparing (laparoscopy or laparotomy)	70	38%	66	39%	
Fertility sparing (method unknown)	9	5%	1	1%	
Non-fertility sparing (method unknown)	9	5%	2	1%	
Unknown	11	6%	12	7%	
Radiotherapy					
No	174	93%	158	94%	0.87
Yes	2	1%	1	1%	
Unknown	11	6%	9	5%	
Chemotherapy					
No	123	66%	113	67%	0.08
Yes	45	24%	48	29%	
Unknown	19	10%	7	4%	
Recurrence					
No	78	42%	104	62%	< 0.001
Yes	70	37%	55	33%	
Unknown	39	21%	9	5%	

#### MOGCT

A total of 42 (91%) of the patients with MOGCT were younger than 50 years at survey (mean 34.1 years, median 31, range 17–70 years). The median age of patients with dysgerminoma was 30 years, with teratoma 37.5 years and with mixed MOGCT 33 years. And, 37 of the 46 patients (80%) underwent fertility-sparing surgery and 26 (57%) received adjuvant chemotherapy. Seven patients (15%) were diagnosed with recurrence (Table 2). Thirty (65%) patients were sexually active and 16 (35%) non-active (Table 3).

**Table 2:** Patient characteristics

At baseline	SCST		MOGCT	
	*n* = 106		*n* = 46	
	*N*	%	*N*	%
Surgical method				
Fertility sparing (laparoscopy)	39	37%	22	48%
Fertility sparing (laparotomy)	9	8%	14	30%
Non-fertility sparing (laparoscopy)	19	18%	3	7%
Non-fertility sparing (laparotomy)	33	31%	3	7%
Fertility sparing (method unknown)	0	0%	1	2%
Non-fertility sparing (method unknown)	2	2%	0	0%
Unknown	4	4%	3	7%
Adjuvant chemotherapy				
No	86.00	81%	20	43%
Yes	18.00	17%	26	57%
Unknown	2.00	2%	0	0%
FIGO stage				
I	87	82%	40	87%
II	6	6%	3	7%
III	4	4%	2	4%
IV	1	1%	0	0%
Unknown	8	8%	1	2%
Age at interview				
< 50 years	33	31%	42	91%
≥ 50 years	73	69%	4	9%
< 1 year	2	2%	2	4%
Time since diagnosis				
1–3 years	15	14%	7	15%
> 3 years	89	84%	37	80%
Recurrence				
No	56	53%	39	85%
Yes	47	44%	7	15%
Unknown	3	3%	0	0%

**Table 3:** Sexual activity and partnership status of patients with sex cord stromal tumors (SCST) or malignant ovarian germ cell tumors (MOGCT)

	No *n*	%	Yes *N*	%	No answer *n*	%
Sexually active						
Entire group	68	45%	83	55%	1	1%
Patients < 50 years	25	33%	50	67%	0	0%
Patients ≥ 50 years	43	56%	33	43%	1	1%
Patients with SCST	52	49%	53	50%	1	1%
Patients with MOGCT	16	35%	30	65%	0	0%
Married or partnership	30	20%	121	80%	1	1%
Change of partner in the last 6 months	147	97%	5	3%		

#### SCST

Patients with SCST were on average 55.7 years old (median 56, range 17 to 86 years). The median age of patients with Granulosa cell tumors was 56 years and with Sertoli–Leydig cell tumors 45 years; 48 patients (45%) underwent fertility-sparing surgery and 18 (17%) received additional chemotherapy. And, 47 (44%) were diagnosed with recurrence (Table 2). And, 53 patients (50%) reported to be sexually active, 52 (49%) to be sexually non-active and one patient did not answer this question (Table 3).

### Sexual activity

Overall, 83 of the participants (55%) reported being currently sexually active; 121 (80%) were married or had a permanent partner (Table 3). Main patient-reported reasons for sexual inactivity were no interest in sex (21%), no partner (17%), a physical problem that makes intercourse unpleasant or difficult (9%), partner has physical problem that makes intercourse unpleasant or difficult (6%) or being too tired (7%) (Table 4). Stratified by histology, patients with MOGCT had an OR of 4.7 (*p* = 0.08) for sexual activity after fertility-conserving surgery compared to non-fertility-sparing surgery (Table 5).

**Table 4:** Reasons for sexual inactivity

Reasons for sexual inactivity	*n*	%
No partner	26	17%
Too tired	11	7%
Partner too tired	5	3%
No interest in sex	32	21%
Partner no interest in sex	7	5%
Physical problem that makes intercourse unpleasant or difficult	14	9%
Partner has physical problem that makes intercourse unpleasant or difficult	9	6%
Other reasons	8	5%

**Table 5:** Association of surgical technique with sexual activity, in the total sample and separately for age and histology groups

Surgery	Not adjusted			Adjusted^a^			
	OR	95% Cl	*p*	OR	95% Cl	*p*	OR
Non-Fertility sparing	1			1			1
Fertility sparing	2.6	(1.3; 5.2)	0.01	1.8	(0.7; 4.7)	0.22	1.8
In women < 50 years old	9.4	(0.9; 99.2)	0.02				
In women ≥ 50 years old	1.3	(0.5; 4.2)	0.59				
Fertility sparing	2.7	(1.4; 5.2)	< 0.01				
In women with SCST	2.3	(1.0; 5.3)	0.04				
In women with MOGCT	4.7	(0.7; 32.6)	0.08				

^a^Adjustment for age, time since diagnosis, FIGO stage, histology, recurrence. In the left part of the table, the crude odds ratios (OR) for fertility-sparing surgery are presented, unadjusted for other variables, for the entire group and separately by age and histology groups

Patients with SCST had 2.3 times the odds of being sexually active when they had received fertility-conserving surgery compared to non-fertility-sparing surgery (*p* = 0.04) (Table 5).

Taken both histology groups together, patients with fertility-conserving surgery had an unadjusted 2.6 fold higher probability for being sexually active than women with non-fertility-conserving treatment (*p* = 0.01). When taking the confounding factors age, FIGO stage, histology and recurrence into account, the odds of being sexually active were still higher for patients after fertility-conserving surgery, but the effect was smaller and the statistical precision lower (OR 1.8, *p* = 0.22) (Table 5). When dividing the unadjusted analysis by age (< 50 or ≥ 50 years), sexual activity after fertility-sparing vs non-fertility-sparing surgery showed an OR of 9.4 (*p* = 0.02) for patients younger than 50 years and an OR of 1.3 (*p* = 0.59) for patients ≥ 50 years (Table 5), although there was no evidence for effect modification using likelihood ratios tests comparing the fully adjusted models.

### Perceived quality of sexual activity

Among those who were sexually active, 35 (42%) reported having high levels of discomfort during intercourse; 38% after fertility-sparing; and 58% after non-fertility-sparing surgery (unadjusted OR 2.2, *p* = 0.11). After adjusting for potential confounders, the odds of having discomfort increased, but the statistical precision again decreased (OR 2.8, *p* = 0.18).

**Fig. 1:**
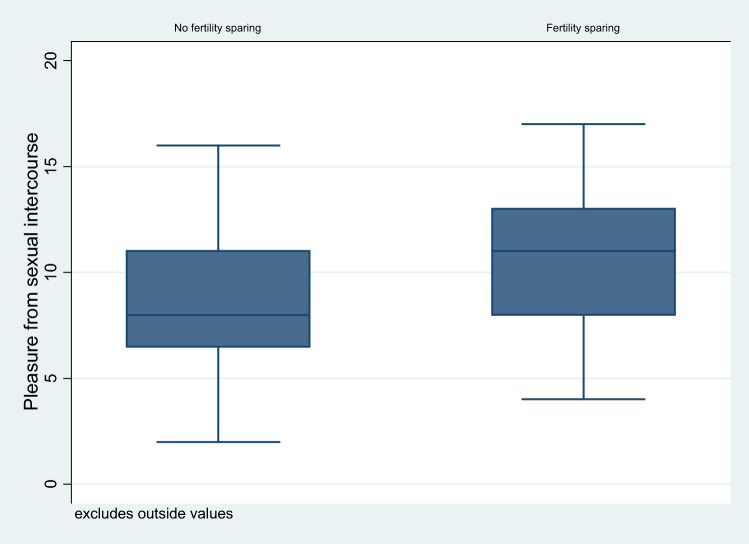
Pleasure during intercourse in association with fertility-sparing or non-fertility-sparing surgery

Women with fertility-conserving treatment had more pleasure with intercourse than women without fertility-sparing surgery (unadjusted *F* 3.3, *p* = 0.07) (Fig. 1). After adjustment for confounding factors, this effect disappeared (*F* 0.4, *p* = 0.52). The largest effect on pleasure in the multivariate model had the FIGO stage (*F* 3.6, *p* = 0.03).

### Global quality of life

**Fig. 2:**
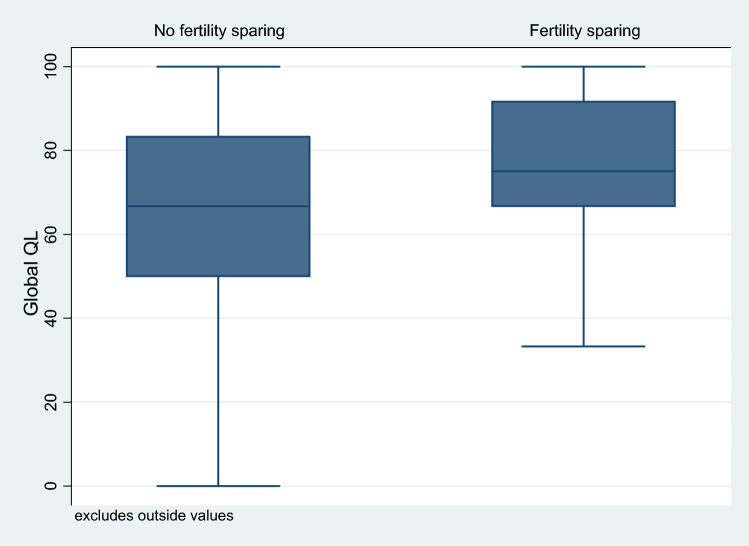
Global quality of life in association with fertility-sparing or non-fertility-sparing surgery

Mean global QoL (gQoL) was 66.1 (scale 0–100, higher values indicating higher QoL) for patients with non-fertility-sparing treatment and 73.8 for women with fertility-conserving surgery (*F* 4.7, *p* = 0.03). After adjustment for confounding factors, there was evidence that QoL was significantly better in the group with the fertility-sparing approach (*F* 2.1, *p* = 0.03) (Fig. 2).

## Discussion

The median age of our responders was 31 years (range 17–70 years) for MOGCT and 56 years (range 17–86 years) for SCST. Of these, 65 and 50%, respectively, were sexually active. The prevalence of sexual activity in SCST patients of CORSETT was comparable to healthy women in the same age group [20]. Sexual activity in our MOGCT patients, however, was only 65% compared to 80% of healthy women in the same age group.

This difference in sexual activity may be explained by the number of non-fertility-sparing surgery in 20% and the administration of chemotherapy in 57% of comparably young patients with MOGCT. Both therapies are known to reduce hormonal function with subsequent influence on sexuality [17, 18].

The main reason for sexual inactivity in our study was “no interest in sex” (20%). Besides other factors, this might reflect the reduced hormonal function.

Patients in our study had an unadjusted 2.7 fold higher probability for being sexually active and a 2.4 lower probability to suffer from discomfort during sexual intercourse after fertility-sparing surgery than after non-fertility-conserving treatment. Non-fertility-sparing surgery was also associated with a significantly reduced global QoL compared to fertility-conserving treatment.

These findings may be the result of several factors like the hormonal ablation after bilateral salpingo-oophorectomy, the extent of surgery or the impact of subsequent chemotherapy [17].

A review on sexual health and cancer of adolescents and young adults reported prevalence rates of sexual problems after oncological treatment ranging from 49 to 43%, within one and two years after diagnosis, respectively [21].

Within a study of long-term survivors “Carolin meets Hanna,” the overall sexuality score for patients with ovarian cancer was much lower with a score of 5.2 than for healthy women of the control group with a score of 28.2 (score range 2.0–36.0 of the FSFI) [22]. Although patients with MOGCT and SCST may not be directly comparable with ovarian cancer patients, the study showed the high prevalence of sexual dysfunction in women after surgery for ovarian tumors.

Therefore, comparably young patients with MOGCT and SCST should be offered prospective counselling to cope with sexual dysfunction caused by disease and/or therapy. Physicians and psycho-oncologists should have a role model function regarding the discussion of these issues. Communication about sexual function, and especially sexual problems, still seems to be difficult for patients and health-care providers, although patients are looking for more information regarding the effects of cancer and of treatment side effects on their sexuality. In a study by Stead et al., although most health-care professionals knew that the majority of patients with ovarian cancer would suffer from a sexual problem, only a quarter of doctors and a fifth of nurses discussed sexual issues with their patients [23]. Reasons for not addressing sexual issues were not feeling responsible, embarrassment in this sensitive and personal area, lack of knowledge and experience, and lack of resources to provide support [23]. In a trial evaluating sexual health as part of gynecologic cancer care, 57% of the patients reported never discussing sexuality [24].

CORSETT implicates that potential long-term consequences on sexual function and quality of life have to be considered for each patient. The potential impact that cancer therapies like surgery or systemic therapy can have on sexual function and fertility needs to be discussed prior to treatment, including preventive options like fertility-sparing surgery, when oncologically safe.

A multi-disciplinary team of oncologists, oncofertility specialists, psycho-oncologists and specialized nurses should be integrated in planning of the therapeutic concept. Shared decision-making for the patient and her partner is of major importance for long-term quality of life [25]. Furthermore, it could be shown for patients with surgery for ovarian cancer that sexual health education and rehabilitation training, relaxation and cognitive training led to significant improvements in overall sexual functioning and psychological distress [26]. Many patients also appreciate if their partner is included in the communication and informed about the possible side effects of cancer and therapy on fertility, sexuality and relationship [27].

The value of our study is to evaluate whether and to which extent non-fertility-sparing surgery impairs sexuality and QoL in patients with MOGCT or SCST. Patients with fertility-sparing surgery had a significantly better QoL, higher sexual activity and less discomfort during intercourse than women with non-fertility-conserving treatment. Therefore, fertility-preserving and sexuality-supporting approaches should be offered to every patient, when oncologically acceptable. Patients with sexual problems or impairment of quality of life after treatment for MOGT or SCST should receive psycho-oncological care and rehabilitation training.

## Data Availability

All data are available from annette.hasenburg@unimedizin-mainz.de.
